# Supplementary material for: Exact reconstruction of gene regulatory networks using compressive sensing
Source: BMC Bioinformatics. 2014 Dec 14;15(1):400. doi: 10.1186/s12859-014-0400-4 (PMC4308013; doi:10.1186/s12859-014-0400-4)
Supplement: Additional file 1 — Supporting Information. [file 12859_2014_400_MOESM1_ESM.docx]

Additional File 1: Supporting Information

*1. Scale of the network*

The proposed method is not limited to the network size itself but it is important to satisfy **Assumption1** and the incoherence condition in order to guarantee exact reconstruction.

In the ideal case with no unobservable species (genes) or noise, the method can be applied to entire networks if the incoherence condition is satisfied (i.e., *n•M ≥ 2s*, practically *n•M ≥ 4s*) where *n* is the number of nodes, *M* is the number of time points and *s* represents the sparsity of the network structure. For example, if we only have one time point measurement (*M=1*), the proposed method cannot be applicable for any networks (*n>2* where *n* is the number of nodes) since it is impossible to satisfy the incoherence condition. As an example, if we consider *n* nodes which are simply connected (i.e., *s=n-1* where *s* is the sparsity of the network), any *2s* columns of an (*n•M)* ×(*n•N)* sensing matrix *Ω* cannot be linearly independent where *N* is the number of the possible candidate basis functions (*N*≫*M*). Thus, **Proposition 1** is not hold and we cannot uniquely reconstruct *s*-sparse signal from *Ω****q.*** However, with a reasonable number of time points (*M*), the incoherence condition could be satisfied.

Similarly, in the practical case with unobservable species (genes) and measurement noise, in order to guarantee the exact recovery of the graph structure, we should satisfy the **Assumption 1** for a given network (hidden node only affect relatively few nodes in the given network) and the incoherence condition. Suppose we choose a small sub-network where a hidden node or unobservable node affects all nodes in the chosen sub-network: then, it is impossible to infer the exact structure of the sub-network. However, if a hidden node only affects relatively few nodes in the given network, we can still reconstruct the exact structure of the sub-network by inferring hidden node influence first..

Therefore, the proposed method is not limited to the network size itself but rather by **Assumption 1** and the incoherence condition. In practice, we can work on a small sub-networks at a time and cover the whole networks by integrating identified sub-networks. Finally, we evaluate computing performance with respect to the size of the problem under various conditions (the number of genes, the number of kinetic features and connectivity) in Additional file 1: Table S1, which provides us a brief guideline of application, e.g. for *n-*genes network with certain kinetic features, how many time points/how much resolution are appropriate for the reconstruction.

*2. Computing performance*

In Equation (12), we construct ***Y*** *=* ***Ωq*** where ***Y*** is an *(n•M)-*dimension vector, ***Ω*** is an *(n•M)×(n•N)* matrix and ***q*** is an *(n•N)-*dimension vector where *n* is the number of genes, *M* is the number of time steps, *N* is the number of possible candidate bases and *S* is the number of nonzero component in ***q***. We evaluate computing performance with respect to various conditions in Additional file 1: Table S1.

| **Network (Size, Linear/Nonlinear, Density)** | | **Parameters** | | | | **Size (Ω) and Sparsity** | | | **L1magic**  **(cpu time [sec])** | **Reconstruction**  **Error** |
| --- | --- | --- | --- | --- | --- | --- | --- | --- | --- | --- |
|  |  | **n** | **N_dic** | **k** | **M** | **n•M** | **n•N** | **S=k•n** |  |  |
| **Small** | Linear | 10 | 1 | 2 | 6 | 80 | 100 | 20 | 0.039977 | 2.7839e-05 |
|  | Nonlinear | 10 | 3 | 3 | 12 | 120 | 300 | 30 | 0.036971 | 2.1576e-05 |
|  | Nonlinear (increase k) | 10 | 6 | 5 | 20 | 200 | 600 | 50 | 0.245067 | 2.5243e-05 |
|  | Nonlinear (many features) | 10 | 9 | 4 | 16 | 160 | 900 | 40 | 0.379294 | 5.4379e-04 |
| **Medium** | Linear | 50 | 1 | 2 | 16 | 800 | 2500 | 100 | 2.021142 | 1.2271e-05 |
|  | Nonlinear | 50 | 3 | 4 | 32 | 1600 | 7500 | 200 | 16.450364 | 1.0452e-05 |
|  | Nonlinear (increase k) | 50 | 3 | 8 | 64 | 3200 | 7500 | 400 | 40.874591 | 3.8697e-06 |
|  | Nonlinear (many features) | 50 | 6 | 8 | 64 | 3200 | 15000 | 400 | 113.578069 | 3.5932e-06 |
| **Large** | Linear | 100 | 1 | 5 | 40 | 4000 | 10000 | 500 | 88.196786 | 1.0844e-06 |
|  | Linear (increase k) | 100 | 1 | 10 | 80 | 8000 | 10000 | 1000 | 272.084944 | 7.4207e-07 |
|  | Nonlinear | 100 | 3 | 5 | 40 | 4000 | 30000 | 500 | 782.021649 | 2.0209e-06 |
|  | Nonlinear (many features) | 100 | 6 | 5 | 50 | 5000 | 60000 | 500 | 2954.001742 | 3.7699e-06 |

**Additional file 1: Table S1**: Computing performance with respect to the size of the problem

(genes, interactions, kinetic features (linear/nonlinearity) and sparse/dense network)

where *n* is the number of genes, *k* is the average connectivity for each node, *N_dic* is the number of candidate bases for each node (i.e., *N = N_dic • n*), and *S* is the sparsity of ***q*** (i.e., *S=k•n*). We choose *M* which satisfies the incoherence condition (*n•M ≥ 4S*) for practical case.

We generate a simple code for comparing computing performance on an application (available online). This code can be used as a guideline for practical application, for example, appropriate parameters study for the reconstruction. Also, this code can be easily modified for real application (TO DO: simply construct ***Y*** and ***Ω*** from time series protein or gene expression data with candidate basis functions). Note that since we are interested in computation time with respect to various conditions (measurements, genes and interactions) here, we simply generate a sensing matrix and observations in the code. Thus, in practical application, computing performance and incoherence condition will depend on biological data set. A simple test code is written in MATLAB R2014a and all experiments were performed on a Macbook Pro with a 2.6GHz dual-core Intel Core i5 processor and 8GB of RAM.

*3. A simple test code for comparing computing performance*

A simple test code is available on the website:

<https://sites.google.com/site/yhchangucb/publications> (bottom page): test_code.m

You may need to download l1-magic code from <http://users.ece.gatech.edu/~justin/l1magic/>

Supporting Figures


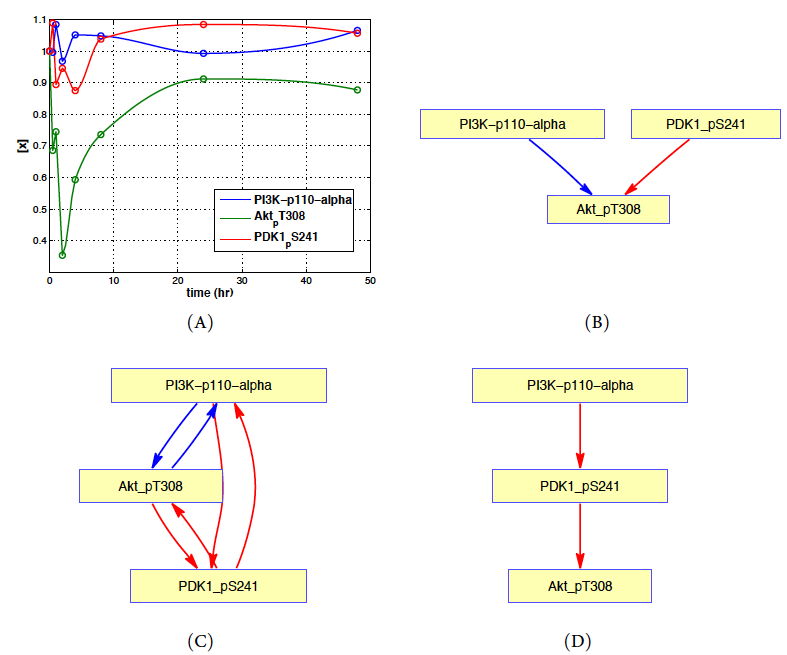


**Additional file 1: Figure S1**. HER2+ overexpressed breast cancer. RPPA dataset (SKBR3 cell line, Serum [32]) (A) gene expression data [0-48hr] (B) L1 optimization result (C) L2 optimization result (D) CS reconstruction result.


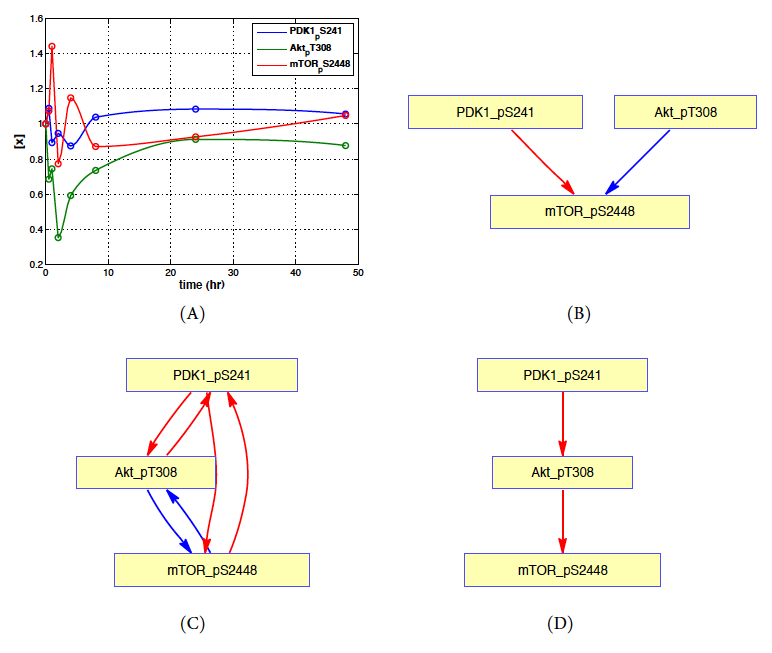


**Additional file 1: Figure S2**. HER2+ overexpressed breast cancer. RPPA dataset (SKBR3 cell line, Serum [32]) (A) gene expression data [0-48hr] (B) L1 optimization result (C) L2 optimization result (D) CS reconstruction result.


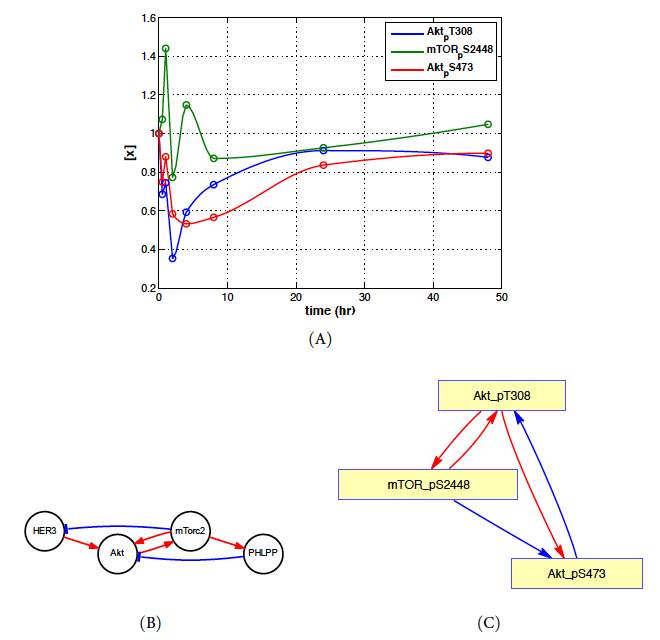
 **Additional file 1: Figure S3**. HER2+ overexpressed breast cancer. RPPA dataset (SKBR3 cell line, Serum [32]) (A) gene expression data [0-48hr] (B) abstract model by M. Moasser [38] (C) CS reconstruction result.
